# P-908. Implementation of Film Array Meningitis/Encephalitis Panel on diagnostic stewardship program in Memorial Hermann Health System, Houston, TX

**DOI:** 10.1093/ofid/ofae631.1099

**Published:** 2025-01-29

**Authors:** Elizabeth A Aguilera, Audrey Wanger, Daniel Moussa, Rodrigo Hasbun

**Affiliations:** UTHealth, McGovern Medical School, Houston, Texas; McGovern Medical School, Houston, Texas; Memorial Hermann Hospital, Houston, Texas; UT Health Mc Govern Medical School, Houston, Texas

## Abstract

**Background:**

The use of multiplex polymerase chain reaction (PCR) panel for detecting the most common pathogens that cause meningitis and encephalitis are increasing and are currently available worldwide. This study aims to evaluate the clinical impact of the implementation of the BioFire Film Array ME (FA ME) panel at Memorial Hermann Health System in Houston, TX.

**Methods:**

This preliminary retrospective study included adults and children with community-acquired meningitis, encephalitis, and cerebrospinal fluid (CSF) WBC > 5 cells/mm3. We categorized the patients into two cohorts: The Pre-Implementation FAME panel-control group (PRE-FAME), which was enrolled from June 2018 to October 2021, compared with the Post-implementation BioFire FilmArray -Intervention group (POST-FAME) evaluated from July 2022 to November 2023.

**Results:**

A total of 440 patients were included (300 in the pre-implementation cohort and 140 in the post-implementation cohort). The majority were male (53%), white (40%), adults (87%) with a median age of 43 years, had a meningitis presentation (79%) and were immunocompetent (75%). There was no significant difference between the PRE-FAME and POST-FAME cohorts (P > 0.05) in sex, age, and immune status, but the post-implementation cohort had a higher proportion of subjects with Charlson Comorbidity Index Score > 1 and more cases with encephalitis (P< 0.05). PRE-FAME patients underwent more singleplex PCR for Herpes Simplex Virus (82% vs. 60%, P< 0.05) and for Varicella zoster virus (42% vs. 7%, P< 0.05). There was a decrease in West Nile Virus (WNV) serologies (37% vs. 20%, P< 0.05) in the POST-FAME cohort. The number of lumbar punctures (LP), CSF cultures, use of Computed Tomography (CT), Magnetic resonance imaging (MRI), duration of antiviral, and length of stay did not differ between the cohorts (P > 0.05). However, there was a reduction in the duration of antibiotic therapy by one day in the POST-FAME cohort and an increase in the diagnostic yield by 10% [(42% in PRE-FAME vs 52% in POST-FAME cohorts (P < 0.05)].

**Conclusion:**

The implementation of the FAME panel increased diagnostic yield, decreased duration of antibiotic use, and decreased the use of single-plex PCR testing and WNV testing but did not impact duration of antiviral therapy or length of stay.

**Disclosures:**

**Rodrigo Hasbun, MD MPH FIDSA**, Biomeriaux: Grant/Research Support|Biomeriaux: Honoraria

